# Pregnancy with Heart Disease: Maternal Outcomes and Risk Factors for Fetal Growth Restriction

**DOI:** 10.3390/ijerph16122075

**Published:** 2019-06-12

**Authors:** Thang Nguyen Manh, Nhon Bui Van, Huyen Le Thi, Long Vo Hoang, Hao Nguyen Si Anh, Huong Trinh Thi Thu, Thuc Nguyen Xuan, Nga Vu Thi, Le Bui Minh, Dinh-Toi Chu

**Affiliations:** 1 Department Obstetrics and Gynecology, Hanoi Medical University, Hanoi 100000, Vietnam; bsnguyenmanhthang@gmail.com (T.N.M.); h.yoga113@gmail.com (H.L.T.); 2 Department of Science and Technology, Hanoi Medical University, Hanoi 100000, Vietnam; 3 Cardiovascular Center, Hanoi Medical University Hospital, Hanoi Medical University, Hanoi 100000, Vietnam; 4 Institute for Preventive Medicine and Public Health, Hanoi Medical University, Hanoi 100000, Vietnam; vohoanglonghmu@gmail.com (L.V.H.); anhhao5896@gmail.com (H.N.S.A.); 5 Department of Dermatology, Dong A Hospital, Hanoi 100000, Vietnam; huong92t@gmail.com; 6 Department of Midwifery, Hanoi Obstetrics and Gynecology Hospital, Hanoi 100000, Vietnam; bs.thucpshn@gmail.com; 7 Institute for Research and Development, Duy Tan University, 03 Quang Trung, Danang 550000, Vietnam; 8 NTT Hi-Tech Institute, Nguyen Tat Thanh University, 300A Nguyen Tat Thanh St., Ward 13, District 4, Ho Chi Minh City 700000, Vietnam; 9 Faculty of Biology, Hanoi National University of Education, Hanoi 100000, Vietnam; 10 School of Odonto Stomatology, Hanoi Medical University, Hanoi 100000, Vietnam

**Keywords:** maternal outcomes, pregnant women, heart disease, risk factors

## Abstract

Caring for children and mothers suffering from cardiac disease is highly challenging, with issues including late diagnosis as well as inadequate infrastructure and supply of drugs. We aimed to evaluate maternal outcomes among pregnant women suffering from heart disease with a live birth, and explored the risk factors for fetal growth restriction among these patients. A retrospective study was performed at the National Hospital of Obstetrics and Gynecology (Hanoi, Vietnam) over a 3-year period from 2014 to 2016. A total of 284 patients were enrolled in the study. Overall, most women were aged below 35 years and were diagnosed with heart disease before pregnancy. Of the women experiencing rheumatic heart disease, the prevalence of mitral valve regurgitation was the highest (40.14%), while the figure for aortic valve regurgitation was the lowest (4.23%). Of women with congenital heart defects, the most common defects were ventricular septal defect (VSD) and atrial septal defect (ASD) (19.37% and 16.55%, respectively), while 5.28% of mothers were diagnosed with tetralogy of Fallot and 1.76% with patent ductus arteriosus. Noted clinical presentations of the patients included palpitation (63.38%), breathlessness (23.59%), leg edema (8.45%), and chest pain (8.1%). The common complications in the study population included 16.90% of women having heart failure and 19.37% having arrhythmias. The incidence of fetal growth restriction was 9.15%. Hypertension (odds ratio (OR): 59.75, 95% confidence interval (CI): 9.1–392.17), the heart disease types (ASD (OR: 4.27, 95% CI: 1.19–15.29) and tetralogy of Fallot (OR: 6.82, 95% CI: 1.21–38.55)), and the complications (heart failure (OR: 10.34, 95% CI: 2.75–38.87) and pulmonary edema (OR: 107.16, 95% CI: 4.96–2313.93)) were observed as risk factors for intrauterine growth restriction. This study provides a cornerstone to promote further studies and to motivate people to apply evidence-based medical care for mothers with diagnosed cardiac disease in the antenatal and postnatal periods.

## 1. Introduction

Heart disease is considered as one of the important concerns resulting in maternal mortality and morbidity in the antenatal and postnatal periods [1]. There are two groups of cardiac disease in women of childbearing age: Congenital and acquired heart disease. The acquired heart disease group comprises rheumatic heart disease (RHD), cardiomyopathies, and ischemic heart disease. Of these, RHD is known as the most common type in developing countries, while cardiomyopathies and congenital heart disease (CHD) are the main types in developed countries. Currently, there has been a reduction in the ratio of RHD and CHD, based on the improvement of early pediatric care and intensive surgical interventions in the childhood [2]. Approximately 0.2–2% of all women in pregnancy are perplexed by heart defects [3], and 20.5% of maternal mortality is associated with cardiac disorders [4].

Compared with developing countries, there are significant differences in health care in developed ones. In developed nations, women can easily access optimal prenatal care together with counselling and solutions to impaired cognition in all centers, but few women suffering from heart disease in developing countries are evaluated and counselled before and during pregnancy [5]. In developed countries, there has been a reduction in the incidence of pregnancies complicated by RHD. However, in developing countries, RHDs are not only still prevalent but continuously a major reason resulting in morbidity and mortality in pregnant mothers [6,7].

Vietnam is listed as a lower middle-income country, where the main source of economy comes from agriculture. According to the reported data from Vietnam’s General Department of Population in early 2018, Vietnam’s population has grown by 1.07% within a year, reaching a total of 93.7 million people. In 2015, the ratio of maternal mortality was 54 per 100,000 live births, and neonatal deaths were in the scale of 18,000. In comparison with developed nations, the care for Vietnamese children and mothers suffering from cardiac disease is much more challenging. In particular, the specific challenges are related to late diagnosis as well as inadequate infrastructure and supply of drugs [8]. Hence, it is important to examine pregnant mothers with cardiac disease in detail to encourage the development of optimal care during pregnancy that becomes an integral part of the overall outcome [9]. However, no formal publication information is available for the maternal outcomes among pregnant Vietnamese women suffering from cardiac disease. It is noticeable that 3%–7% of all newborns are affected by fetal growth restriction, which is associated with many adverse outcomes including stillbirth [10], neonatal mortality [11], hypoxic–ischaemic encephalopathy [12], special educational needs [13], and many other health problems in their adulthood [14]. Therefore, the findings for the associated risks of fetal growth restriction need to be considered. The purpose of this work is to evaluate the maternal outcomes among pregnant Vietnamese women experiencing cardiac disease with a live birth, and to explore the risk factors for fetal growth restriction in these women.

## 2. Methods

### 2.1. Study Design

We designed a retrospective study which was performed at the National Hospital of Obstetrics and Gynecology (NHOG) (Hanoi, Vietnam) in the 3-year period from 2014 to 2016. Permission to enter the NHOG was obtained from the ethics board of the NHOG. Data from this retrospective study were only for research purposes. This study did not contain any interference to patients.

The study population was established by pregnant mothers after 22 weeks of gestation, with diagnosed heart disease and being treated at the NHOG during the time surveyed, and having a live birth in this pregnancy. We excluded the records of women: (i) Suffering from other medical conditions (such as glomerulonephritis, renal failure, basedow, diabetes mellitus, liver disease, pulmonary disease, hyperlipoproteinemia, uremia, and pre-eclampsia) and (ii) having fetal outcomes (e.g., stillbirth, prematurity, and disability). The sample size formula for the quantitative variable was as follows:

(1) $$n=Z_{1-\frac{\alpha }{2}}^{2}\;.\;\frac{p\;.\;q}{{\left(p\;.\;\varepsilon \right)}^{2}}$$

Here, Z_(1−α/2)_ = 1.96 with α = 0.05; p = 0.93, which was recorded as the prevalence of pregnant women suffering from heart disease who were dealt with by obstetrics (vaginal delivery, caesarean delivery, and forceps) [15]; and ε = 0.032 was the margin of error. The sample size from the above formula was calculated as 282. 284 patients were enrolled in this study.

### 2.2. Variables

The primary outcome measure was fetal growth restriction, defined as the weight at birth being less than the 10th centile, based on a modified centile chart [16].

The selected independent variables included maternal age (<35 and ≥35 years of age), time of heart disease diagnosis (before and after pregnancy), gravida (primi, gravida 2, and gravida 3, and more), clinic presentation (no/yes), hypertension (no/yes), atrial septal defect (ASD; no/yes), ventricular septal defect (VSD; no/yes), tetralogy of Fallot (no/yes), heart failure (no/yes), pulmonary edema (no/yes), and heart arrhythmia (no/yes).

### 2.3. Statistical Analysis

All data were analyzed with Stata^®^ 15.0 (Stata Corporation, College Station, TX, USA). A *p*-value of <0.05 was considered statistically significant. Descriptive statistics were applied to characterize the prevalences of types of heart disease, clinical presentations, cardiac complications, and functional classes of cardiac disease on the NYHA (New York Heart Association) classification. All descriptive figures were presented with the usage of Python 3.7.2-programming language (Python Software Foundation) (https://www.python.org/about/). The selected risk factors for fetal growth restriction were detected with a multivariate model, using multiple logistic regression analysis. We first ran the univariate analyses (i.e., relation of the outcome ‘fetal growth restriction’ with each explanatory variable), and then used only those variables which met a preset cutoff for significance to run a multivariable model. This cutoff with *p* < 0.10 was applied to identify potential associated variables, rather than to test a hypothesis. In particular, we conducted the different models, and then a model with maximum parsimony was recorded and used for the present paper (minimum number of risk factors and maximum discriminatory power).

### 2.4. Ethical Approval

All procedures performed in studies involving human participants were in accordance with the ethical standards of the institutional and/or national research committee and with the 1964 Helsinki declaration and its later amendments or comparable ethical standards. This study was approved by the ethics board of the NHOG (decision number 820/CN-PSTW, issued on October 14, 2017). Informed consents were obtained from all individual participants included in the study.

## 3. Results

The proportion of pregnant mothers diagnosed with cardiac disease in the age group of below 35 years (86.97%) was much higher than that of mothers aged 35 years and over (13.03%). The mean age was 28.18 years (SD (standard deviation): 5.05; range: 18 to 44). The percentage of patients with gravida 3 or more was the lowest, at only 9.86%, while the figures for pregnant women for both the first time and second time were above 42%. Most women with heart disease were diagnosed before pregnancy (80.63%). While the percentage of women working in an office was 39.44%, the figures for workers and other careers were 13.38% and 47.18%, respectively. The proportion of women living in rural areas (55.28%) was higher than that of women living in urban areas (44.72%) (Table 1).

**Figure 1:**
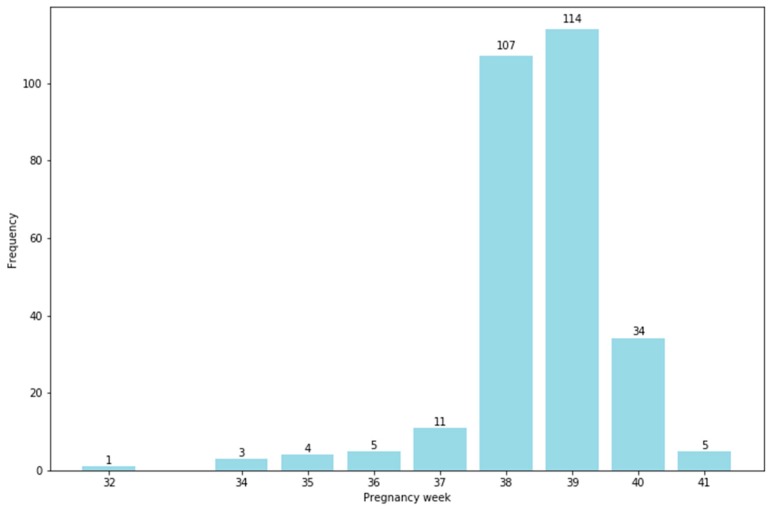
Gestational week at the time of delivery.

**Figure 2:**
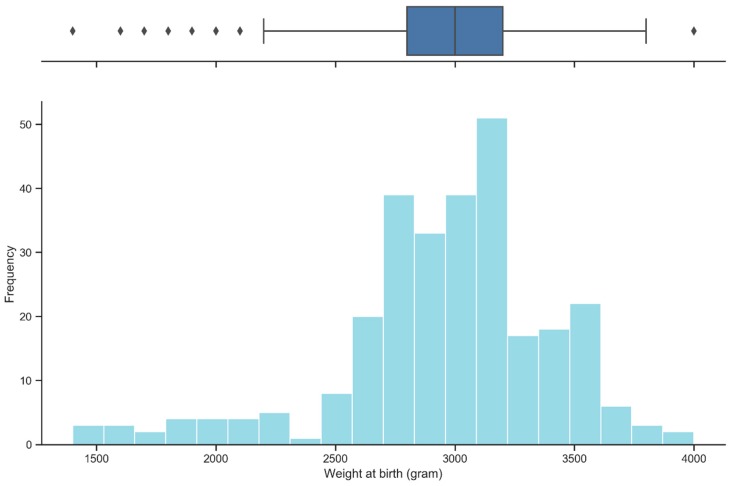
Babies’ weights at birth (*n* = 284).

Most mothers gave birth to their infants at 38 and 39 weeks (37.68% and 40.14%, respectively). The latest time of delivery recorded was 41 weeks, with five cases (1.76%), while only one case was delivered at 32 weeks (0.35%). The mean gestational age at the time of delivery was 38.51 (SD: 1.14; range: 32 to 41) (Figure 1). Most of the pregnancies were full-term (37 to 42 weeks) (95.42%). Most infants weighed 2500 grams or more (90.85%) (Figure 2).

As shown in Table 2, of women with RHD, the prevalence of mitral valve regurgitation was the highest (40.14%), while the figure for aortic valve regurgitation was the lowest (4.23%). Of women with CHD, the prevalences of individuals suffering from VSD and ASD were high, at 19.37% and 16.55%, respectively, while 5.28% of mothers were diagnosed with tetralogy of Fallot and 1.76% with patent ductus arteriosus.

**Figure 3:**
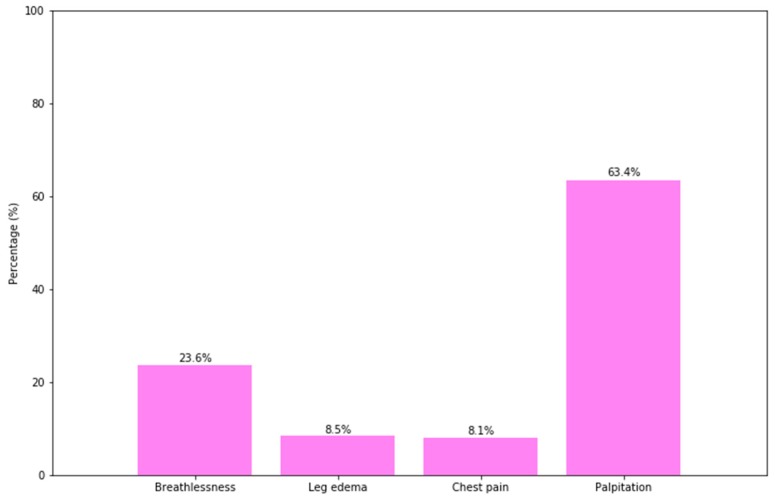
Clinical presentations of the patients (*n* = 284).

The most common clinical presentation among pregnant women was palpitation (63.38%). The proportion of individuals with breathlessness was the second rank, at 23.59%, while the figures for leg edema and chest pain decreased in order, at 8.45% and 8.1%, respectively (Figure 3). As shown in Table 3, it was found that common complications in the study population included 16.90% of women having heart failure and 19.37% having arrhythmias. There were only four cases of pulmonary edema, with a proportion of 1.41%.

Of 284 heart disease patients, the number of patients suffering from heart failure was 48. For the functional class of cardiac disease on NYHA classification, of the 48 women experiencing heart failure, individuals with grade I (31.25%) and grade II (50%) disease were common. While the figure for grade III was 16.67%, the lowest prevalence was observed among mothers with grade IV, with only one case (Table 4).

The total number of cases of fetal growth restriction recorded was 26 (9.15%). Table 5 presents the associated factors of fetal growth restriction among mothers suffering from heart disease in multivariate logistic progression models. There was a remarkable association between hypertension and fetal growth restriction. Women with hypertension had significantly higher odds of fetal growth restriction than others who had not suffered from hypertension (odds ratio (OR): 59.75, 95% confidence interval (CI): 9.1–392.17). The odds ratio of fetal growth restriction was 4.27 (1.19–15.29) in ASD and 6.82 (1.21–38.55) in tetralogy of Fallot (*p* < 0.05). The odds of fetal growth restriction were significantly higher in women experiencing heart failure than others (OR: 10.34, 95% CI: 2.75–38.87). This model indicated the noticeably positive association of fetal growth restriction with women having complications of pulmonary edema (OR: 107.16, 95% CI: 4.96–2313.93).

## 4. Discussion

In this study, the patients who suffered from heart disease were treated according to the cardiovascular treatment regimen (the specific treatment for each case), which depended on the time of disease detection, the time of the patient arriving at the hospital, and the specific clinical condition of each case. Nevertheless, specific treatments for each case are not presented in this paper due to this content not being included in the primary research objectives.

We found out the various cardiac lesion types in all patients and conducted the evaluation of maternal outcomes in pregnant patients diagnosed with cardiac disease. Most of the pregnant women with heart disease were aged below 35 years and were diagnosed before pregnancy. In fact, mothers could get important information about pregnancy-related heart disease in their lives through the consults/recommendations/requests from doctors as well as through the mass media around them. Hence, women were perhaps afraid of getting pregnant at an increasing age in the background of their own heart disease, or their pregnancies were considered carefully after clearly understanding the occurred and/or possible consequences. Pregnancy in a mother suffering from cardiovascular disease causes significant changes in cardiovascular and hemodynamic health, which may result in obstetric and neonatal complications. Heart disease in pregnancy encompasses a wide spectrum of disorders. In Vietnam, maternal deaths from valvular heart disease are increasing and often happen in people with no history of heart disease. We found that, despite mothers suffering from heart disease, most of their pregnancies are full-term (the term “pregnancy” was defined as a live birth from 37 completed weeks to less than 42 completed weeks of gestation [17]). Furthermore, in this study, overall, RHD was much more common than CHD, which was consistent with previous studies [18,19,20,21]. The explanation for this can be due to the lack of preventive treatment and inadequate use of secondary antibiotic prophylaxis against streptococcal infections in Vietnam. We found that mitral valve regurgitation was predominantly due to valvular lesions (40.12%). However, most previous studies indicated that mitral valve stenosis was more common [15,18,19,20,21,22]. The reason might be that our study only concentrated on pregnant mothers after 22 weeks of gestation, whereas others included mothers over the whole pregnancy period. The prevalences of types of valvular lesions, therefore, were clearly dissimilar. Among women with CHD, VSD and ASD were observed to be the most common in our study.

Noted clinical presentations of patients included palpitation (63.38%), breathlessness (23.59%), leg edema (8.45%), and chest pain (8.1%). Three of these presentations (except for chest pain) were also reported in a study of Abbas (Bangladesh) [20]. Our study as well as Abbas’s indicated that palpitation and breathlessness were common symptoms in pregnant women suffering from cardiac disease. It is noteworthy that the figure for palpitation in our study was twice as high as Abbas’s (27.5%), but our figure for the clinical presentation of breathlessness (23.59%) is only half of Abbas’s (41.2%). Although Bangladesh and Vietnam are both lower middle-income countries, perhaps the differences in living and working patterns lead to varying rates in the appearances of these symptoms. Obviously, this paper was more meaningful to extrapolate to the population since our result had a larger sample size compared to Abbas’s one, which had a total of 51 pregnant women with diagnosed heart disease.

Pregnant women with heart disease will experience changes in their heart and blood vessels, which increase the burden on heart muscle. In our study, it was found that the complications of heart failure and arrhythmias were common in the study population, at around 18%, whereas the prevalence of mothers with pulmonary edema was 1.41% (four cases). In particular, the mothers with pulmonary edema did not receive any type of fluid administration during the study period. Interventions for patients were only conducted promptly as soon as complications occurred or clinical presentations got worse. Our result was totally consistent with a finding in the study of Pillutla et al. [18]. They found that 31% of cases experienced a cardiovascular event, and the cardiac events of either heart failure or arrhythmias were the most prevalent among pregnant women [18]. Our result was also in line with Joshi’s study, which reported that heart failure was considered as a common complication among 42 women with cardiac disease [19]. However, the complications including arrhythmias and pulmonary edema were not recorded in Joshi’s study. Despite both retrospective studies, the differences of recorded complications might be due to the significantly different sample sizes between the studies. Notably, the complication of heart failure is always considered to be a major global problem, as it influences at least 26 million people worldwide and its rate is growing [23]. The economic burden of heart failure is significant for many countries, particularly in developing countries such as Vietnam [23]. Out of all the cardiac disease patients in our study, there were only 48 cases of heart failure, with a proportion of 16.90%. Pregnant women who were classified into NYHA class I and II were more common. Similar findings were also observed in the previous studies [19,24,25].

Pregnant women with cardiovascular disease that has not been detected are at a high risk of adverse maternal and fetal outcomes. Most women in our study had full-term pregnancies with normal weights at birth (90.85%) as defined by the World Health Organization, regardless of gestational age. Nevertheless, a form of fetal growth restriction was also recorded as a common unexpected consequence. In particular, the incidence of fetal growth restriction after 22 weeks of gestation in Vietnamese women with heart disease was 9.15%. In Vietnam, the incidence of newborns experiencing intrauterine growth restriction was 11%, while this figure was around 8% in Indonesia, Malaysia, and Thailand, and 6% in the People’s Republic of China [26]. A retrospective analysis of clinical data obtained from mothers and newborns in Mainland China showed that the incidence rate of fetal growth restriction was 8.77%. The fetal growth restriction incidence of 9.15% is not low, because we only concentrated on pregnant women suffering from cardiac disease after 22 weeks of gestation. Most other studies were based on data obtained from mothers and newborns. In addition, a publication based on animal models suggested that the proportion of fetal growth restriction was up to 8% of pregnancies [27], which was lower than the figure of 9.15% in the present study.

Intrauterine growth restriction, a dominant health issue in the neonatal period, is a condition in which a fetus is incapable of reaching its normal physical size. We conducted this study to explore the risk factors for fetal growth restriction among mothers with heart disease, which were considered by a multivariate logistic progression model with maximum parsimony. Our results indicated that there were noticeable associations between time of hypertension and growth restriction of the fetus. Mothers diagnosed with hypertension had higher odds of fetal growth restriction than women without hypertension (OR: 59.75, 95% CI: 9.1–392.17). Our result was compatible with a retrospective cohort study exploring the relationships between pregnancy-induced hypertension types and fetal growth. This study presented that pregnancy-induced hypertension, especially preeclampsia, had a significant impact on intrauterine growth restriction [28]. Thus, hypertension among pregnant mothers in our study was also found as a substantial factor increasing the risk of fetal growth restriction in pregnant mothers. In particular, this study only concentrated on women suffering from heart disease, so the independent variable of hypertension for fetal growth was essential. As was shown in previous studies, intrauterine growth restriction has been attributed to various maternal factors, in particular maternal vascular disease which accounts for 20–30% of all intrauterine growth restriction related to decreases in uteroplacental perfusion [29,30]. In the present study, we found that the odds ratio of fetal growth restriction was 4.27 (1.19–15.29) in ASD and 6.82 (1.21–38.55) in tetralogy of Fallot (*p* < 0.05), which suggested the significant effects of ASD and tetralogy of Fallot on fetal growth. This knowledge is necessary for physicians and clinical specialists to consider the interventions in order to reduce the risk of fetal growth restriction, which may not only result in perinatal morbidity and death but is also a known risk factor for cardiovascular disease and metabolic syndrome in later life. In addition, our results suggested that specific complications had clear associations with intrauterine growth restriction [31]. As was given in the Results section, the odds of fetal growth restriction was significantly higher in women experiencing heart failure than others (OR: 10.34, 95% CI: 2.75–38.87). Our regression model indicated the significantly positive association of fetal growth restriction with women having the complication of pulmonary edema (OR: 107.16, 95% CI: 4.96–2313.93). Notably, the odds rates of fetal growth restriction were remarkably plausible for the above risk factors. This could be due to a combination of different factors, especially that Vietnam is a developing country in Asia where a large number of intrauterine growth restriction infants are seen [32]. Although we were unable to control for all confounding factors that influence both fetal growth restriction and the reported-associated factors, our data suggested that the current findings will help specialists consider reported factors to reduce the rate of fetal growth restriction in mothers with heart disease.

This is the first study to concentrate on maternal outcomes in pregnant mothers (after 22 weeks of gestation) who experienced cardiac disease and were reported with a live birth in the present pregnancy, and had risk factors for fetal growth restriction in this nation. Neonatal outcomes (e.g., stillbirth, prematurity, and disability) were excluded to limit the factors that might act as interfering or interacting factors towards intrauterine growth restriction among mothers with heart disease with a live birth. There are several limitations in the present study which need pointing out. Despite having searched for a model with maximum parsimony (minimum number of risk factors and maximum discriminatory power), we did not adequately explore maternal, placental, fetal, or genetic factors resulting in intrauterine growth restriction of women, because some of the data was not adequately included in the clinical record. This retrospective study design did not allow us to determine any causal relations between risk factors and fetal growth restriction. Since patients’ medical records from the hospital used in this retrospective study only included complications’ labels that were reported by specialists, we could not take into account the onsets of the occurred complications. Cardiac functional parameters were not included in the collection process of the retrospective data from medical records, and should be considered in further studies. Gravidity and parity in medical records were based on self-reported information from mothers with heart disease, and the information provided by them was not validated. Another limitation of this study was that we did not assess the number of mothers with spontaneous pregnancies and/or induced pregnancies.

## 5. Conclusions

This study supported the fact that the major influence on pregnancy outcomes in women is existing heart disease. The incidence of fetal growth restriction after 22 weeks of gestation was recorded to be 9.15% of all study women. Notably, hypertension, the heart disease types (ASD and tetralogy of Fallot), and the complications (heart failure and pulmonary edema) were found as important risk factors for intrauterine growth restriction among pregnant mothers suffering from heart disease. The present data provides important evidence for physicians and clinical specialists for treatment considerations as well as appropriate interventions. This study will also provide a cornerstone to promote further studies and to motivate people to apply evidence-based medical care during pregnancy for mothers diagnosed mothers cardiac disease.

## Figures and Tables

**Table 1:** Some selected characteristics of study patients (*n* = 284).

Characteristics	*n*	%
**Age group**		
<35	247	86.97
≥35	37	13.03
Mean (SD *; Min–Max)	28.18 (5.05; 18–44)	
**Gravida**		
Primi	135	47.54
Gravida 2	121	42.61
Gravida 3 or more	28	9.86
**Diagnosis time**		
Before pregnancy	229	80.63
After Pregnancy	55	19.37
**Occupation**		
Officer	112	39.44
Worker	38	13.38
Others	134	47.18
**Area**		
Urban	127	44.72
Rural	157	55.28

*: standard deviation.

**Table 2:** Types of cardiac disease among study patients (*n* = 284).

The Types of Heart Disease	*n*	%
**Rheumatic heart disease**		
Mitral valve stenosis	65	22.89
Mitral valve regurgitation	114	40.14
Mitral stenosis + Mitral valve regurgitation	46	16.20
Aortic valve regurgitation	12	4.23
Pulmonary stenosis	17	5.99
**Congenital heart disease**		
ASD	47	16.55
VSD	55	19.37
Tetralogy of Fallot	15	5.28
Patent ductus arteriosus	5	1.76

**Table 3:** Cardiac complications of the patients (*n* = 284).

Complications	*n*	%
Heart failure	48	16.90
Pulmonary edema	4	1.41
Heart arrhythmia	55	19.37

**Table 4:** Functional class of heart disease according to New York Heart Association (NYHA) classification (*n* = 48).

NYHA Classification	*n*	%
I Grade	15	31.25
II Grade	24	50.00
III Grade	8	16.67
IV Grade	1	2.08

**Table 5:** Related factors of fetal growth restriction among pregnant mothers with heart disease: Multivariate logistic progressions (*n* = 284).

Characteristics	FRG (*n*, %)	Without FRG (*n*, %)	*p*-Value	Fetal Growth Restriction (26, 9.15%)	
				OR	95% CI
**Age of mother**					
<35	21 (8.50%)	226 (91.50%)	0.356 ^F^	1	-
≥35	5 (13.51%)	32 (86.49%)		1.08	0.24–4.84
**Time diagnosed with heart disease**					
Before pregnancy	15 (6.55%)	214 (93.45%)	0.002 *^p^*	1	–
After pregnancy	11 (20.00%)	44 (80.00%)		1.84	0.6–5.62
**Gravida**					
Primi	13 (9.63%)	122 (90.37%)	0.164 ^F^	1	-
Gravida 2	8 (6.61%)	113 (93.39%)		0.62	0.19–2.02
Gravida 3 or more	5 (17.86%)	23 (82.14%)		0.91	0.2–4.24
**Clinic presentation**					
No	8 (8.42%)	87 (91.58%)	0.831 *^p^*	1	-
Yes	18 (9.52%)	171 (90.48%)		0.59	0.19–1.8
**Hypertension**					
No	22 (7.97%)	254 (92.03%)	0.003 ^F^	1	-
Yes	4 (50.00%)	4 (50.00%)		59.75 ***	9.1–392.17
**Congenital heart diseases**					
**ASD**					
No	19 (8.02%)	218 (91.98%)	0.135 *^p^*	1	-
Yes	7 (14.89%)	40 (85.11%)		4.27 *	1.19–15.29
**VSD**					
No	23 (10.04%)	206 (89.96%)	0.435 ^F^	1	-
Yes	3 (5.45%)	52 (94.55%)		0.28	0.05–1.7
**Tetralogy of fallot**					
No	23 (8.55%)	246 (91.45%)	0.148 ^F^	1	-
Yes	3 (20.00%)	12 (80.00%)		6.82 *	1.21–38.55
**Complications**					
**Heart failure**					
No	15 (6.36%)	221 (93.64%)	0.000 *^p^*	1	-
Yes	11 (22.92%)	37 (77.08%)		10.34 **	2.75–38.87
**Pulmonary edema**					
No	23 (8.21%)	257 (91.79%)	0.003 ^F^	1	-
Yes	3 (75.00%)	1 (25.00%)		107.16 **	4.96–2313.93
**Heart arrhythmia**					
No	21 (9.17%)	208 (90.83%)	1 ^F^	1	-
Yes	5 (9.09%)	50 (90.91%)		2.65	0.58–12.17

Note: FRG: Fetal growth restriction; CI: Confidence interval; OR: Odds ratio; *p*: Pearson chi-square test, F: Fisher’s exact test; * *p* < 0.05, ** *p* < 0.01.
